# Supplementary material for: Association of First-Trimester Maternal Biomarkers with Preeclampsia and Related Maternal and Fetal Severe Adverse Events
Source: Int J Mol Sci. 2025 Jul 11;26(14):6684. doi: 10.3390/ijms26146684 (PMC12295517; doi:10.3390/ijms26146684)
Supplement: Supplementary file 1 [file ijms-26-06684-s001.zip › ijms-3640462-supplementary.pdf]

International Journal of
Molecular Sciences

Supplementary Materials

Biomarkers methodology

sFlt-1 and PlGF levels were determined using electrochemiluminescence immunoassays (ECLIA) on the automated Cobas 8000 analyzer (Roche Diagnostics, Penzberg, Germany). The sFlt-1/PlGF ratio was then calculated.

Total cell-free DNA (cfDNA), both fetal and maternal, was measured from the sera using the QIAamp Blood Mini Kit (Qiagen, UK). Quantification of cfDNA was performed using qRT-PCR, amplifying a segment of the constitutive beta-globin gene. Specific primers and Taqman® probes, as well as the CFX96 RT-PCR system (Bio-Rad), were used for this purpose.

Microvesicles (MVs) were determined using flow cytometry (BD LSR Fortessa; BD Biosciences). Flow counts (Beckman Coulter, Marseille, France) were added in both cases to determine the number of MVs per µL of serum. Centrifugation of 250 µL of serum was carried out for 20 min at 3500 G at room temperature to obtain a platelet-free serum sample; after that, samples were centrifuged for 30 min at 17.000 g at room temperature twice to obtain an MV pellet. All samples were frozen and stored at -80 °C until assayed. MVs were identified as events with a 0.1-1 µm diameter on forward light scatter (FSC) and side-angle light scatter (SSC) intensity dot plot representation, by comparison to flow cytometry calibration beads (Megamix-plus side angle light scatters SSC [0.16, 0.20, 0.24 and 0.5 µm], Biocytex, Marseille, France), indicating that our MV isolation strategy mainly detected large vesicles or microvesicles. The serum was incubated with fluorochrome-labeled antibodies to determine cellular origin. Fifty µL of processed plasma was incubated with 3 µL of a monoclonal antibody anti-human CD31 FITC and CD41 Pacific blue to differentiate between CD31+CD41+ platelet MVs (PMVs) and CD31+CD41− endothelial MVs (EMVs). Total microvesicles were identified by annexin V positivity (AV^+), while endothelial MV were AV^+, $CD31^+$ and $CD4^-$ and platelet MV were AV^+, $CD31^+$ and $CD41^+$. Data represent the mean of two independent measures.

Beta human chorionic gonadotropin (β-hCG) and pregnancy-associated plasma protein A (PAPP-A) concentrations were determined using an electrochemiluminescence technique on a Cobas 6000 analyzer (Roche Diagnostics Penzberg, Germany).

Statistical analysis

Categorical variables are presented as absolute and relative frequencies (n, %). Quantitative variables are shown as mean ± standard deviation (SD) when they followed a normal distribution, or as median and interquartile range (IQR) in case of non-normally distributed data. Normality of distributions was assessed using the Kolmogorov–Smirnov test. For the comparison of categorical variables, the chi-square test (or Fisher's exact test where appropriate) was used. To compare quantitative variables, Student's t-test or one-way ANOVA (for comparison of 2 or ≥3 groups, respectively) was applied for normally distributed variables; the Mann–Whitney U test or the Kruskal–Wallis test was used for non-normally distributed variables. Post hoc analysis was performed using the Bonferroni test. The correlation between quantitative variables was evaluated using Pearson's or Spearman's correlation test, as appropriate.

Supplementary tables

Table S1. Association between biomarkers and serious adverse events.

	**OR (95%CI), *p*-value**
**PLGF**	

**OR fetal events**	
≤36 pg/ml	1 (ref)
>36 pg/ml	**0.253 (95%CI 0.093, 0.686), $p = 0.007$**
**OR maternal events**	
≤36 pg/ml	1 (ref)
>36 pg/ml	1.812 (95%CI 0.377, 8.718), $p = 0.458$
**OR maternal and fetal events**	
≤36 pg/ml	1 (ref)
>36 pg/ml	**0.266 (95%CI 0.101, 0.702),fig$p = 0.008$**
**cfDNA**	
**OR fetal events**	
≤1162 ng/ml	1 (ref)
>1162 ng/ml	1.035 (95%CI 0.450, 2.384), $p = 0.935$
**OR maternal events**	
≤1162 ng/ml	1 (ref)
>1162 ng/ml	**8.057 (95%CI 0.928, 69.987), $p = 0.059$**
**OR maternal and fetal events**	
≤1162 ng/ml	1 (ref)
>1162 ng/ml	1.389 (95%CI 0.601, 3.209), $p = 0.442$
**sFLT1**	
**OR fetal events**	
≤1858 pg/ml	1 (ref)
>1858 pg/ml	0.842 (95%CI 0.310, 2.288), $p = 0.736$
**OR maternal events**	
≤1858 pg/ml	1 (ref)
>1858 pg/ml	**29.143 (95%CI 3.249, 261.379), $p = 0.003$**
**OR maternal and fetal events**	
≤1858 pg/ml	1 (ref)
>1858 pg/ml	1.544 (95%CI 0.562, 4.246), $p = 0.400$
